# Importance and determinants of Gleason score undergrading on biopsy sample of prostate cancer in a population-based study

**DOI:** 10.1186/1471-2490-13-19

**Published:** 2013-04-11

**Authors:** Elisabetta Rapiti, Robin Schaffar, Christophe Iselin, Raymond Miralbell, Marie-Françoise Pelte, Damien Weber, Roberto Zanetti, Isabelle Neyroud-Caspar, Christine Bouchardy

**Affiliations:** 1 Geneva Cancer Registry, Institute for Social and Preventive Medicine, University of Geneva, 55 boulevard de la Cluse, 1205 Geneva, Switzerland; 2 Division of Urology Surgery, Geneva University Hospitals, rue Gabrielle Perret-Gentil 4, 1211 Geneva 14, Switzerland; 3 Division of Radiation Oncology, Geneva University Hospitals, avenue de la Roseraie 53, 1205 Geneva, Switzerland; 4 Division of Clinical Pathology, Geneva University Hospitals, rue Michel-Servet 1, 1206 Geneva, Switzerland; 5 Piedmont Cancer Registry, CPO, Torino, Italy

**Keywords:** Prostate cancer, Gleason score, Biopsy, Prostatectomy, Population-based study, Biopsy undergrading

## Abstract

**Background:**

In this population-based study, we investigated the degree of concordance between Gleason scores obtained from prostate biopsies and those obtained from prostatectomy specimens, as well as the determinants of biopsy understaging.

**Methods:**

We considered for this study all 371 prostate cancer patients recorded at the Geneva Cancer Registry diagnosed from 2004 to 2006 who underwent a radical prostatectomy. We used the kappa statistic to evaluate the Gleason score concordance from biopsy and prostatectomy specimens. Logistic regression was used to determine the parameters that predict the undergrading of the Gleason score in prostate biopsies.

**Results:**

The kappa statistic between biopsy and prostatectomy Gleason score was 0.42 (p < 0.0001), with 67% of patients exactly matched, and 26% (n = 95) patients with Gleason score underestimated by the biopsy. In a multi-adjusted model, increasing age, advanced clinical stage, having less than ten biopsy cores, and longer delay between the two procedures, were all independently associated with biopsy undergrading. In particular, the proportion of exact match increased to 72% when the patients had ten or more needle biopsy cores. The main limitation of the study is that both biopsy and prostatectomy specimens were examined by different laboratories.

**Conclusions:**

The data show that concordance between biopsy and prostatectomy Gleason scores lies within the classic clinical standards in this population-based study. The number of biopsy cores appears to strongly impact on the concordance between biopsy and radical prostatectomy Gleason score.

## Background

The Gleason grading system, based upon architectural features of prostate cancer cells, is the most widely used histological grading method for prostatic adenocarcinoma. The Gleason score (GS) closely correlates with clinical behavior, and provides an important index of prognosis. Furthermore, this score is one of the key determinants in treatment decision making, together with stage, age and prostate-specific antigen [1]. Indeed, most models who were developed to predict the likelihood of disease extension beyond the prostate gland, and to correlate pre-treatment findings with long-term outcomes, incorporate biopsy GS [1].

Significant discrepancies exist between the GS determined by the prostate biopsy, and the GS based upon the pathologic specimen. It has been observed that the GS from needle biopsies underestimates the GS of the radical prostatectomy specimen in 19 to 57% of all cases depending on the series and the periods examined [2-8]. Such downgrading has a significant impact on treatment decisions and patient outcomes, particularly when the choice of treatment is between active surveillance and curative intent therapy.

Several factors can influence the likelihood that the biopsy GS underestimates the prostatectomy score, including the PSA level, the level of pathologist expertise, the patient’s age, the results of the digital rectal examination, the prostate gland volume, the percentage of cancer cells in the biopsy sample and the number of biopsies obtained [5,7,9-11]. With reference to the last issue, it is worth mentioning that the European Association of Urologists (EAU) in 2008 recommended obtaining at least 10 cores during the biopsy [12], while in the most recent guidelines reduced this number to eight [13].

In this population-based study, we investigated the degree of concordance between GSs obtained from needle biopsy of the prostate and radical prostatectomy, and we assessed the parameters that may be related to undergrading the GS in prostate biopsy.

## Methods

Using data from the population-based Geneva Cancer Registry, we considered for this study all 371 prostate cancer patients diagnosed from 2004 to 2006, who underwent a radical prostatectomy.

The Geneva Cancer Registry collects information from various sources and is considered accurate, as witnessed by its very low percentage (<2%) of cases recorded from death certificates only [14]. All hospitals, pathology laboratories, and private practitioners in the canton of Geneva are requested to report every cancer case. Trained tumor registrars systematically extract data from medical and laboratory records and physicians regularly receive enquiry forms to complete missing data.

Recorded data for each cancer case include sociodemographic variables; method of detection; prostate-specific antigen (PSA) value at diagnosis; tumor characteristics (including histology, differentiation based on tumor grade, and GS); stage at diagnosis according to both clinical and pathological TNM classification; lymph node status; treatment; survival status; and cause of death. Different laboratories, from both the public and the private sector, examine biopsy and prostatectomy specimens in Geneva. For this study, the variables of interest were age, socioeconomic status based on the patient’s last occupation (high, middle, low, unknown), sector of care of the first treatment (public, private), PSA value at diagnosis (<10 ng/ml, 10–20, >20) and clinical stage (T1-T2, T3-T4, Tx). For each patient, we retrieved information on the number of biopsy cores and the volume of the prostate in cubic centimeters (cm^3^). We also calculated the number of biopsy procedures performed by each operator during the study period within the study population as an indicator of the experience of the biopsy performers. To account for the possibility of a change in the severity of the disease occurring during the delay between the biopsy and the prostatectomy, we calculated the difference in days between the prostatectomy and the biopsy dates.

Concordance between biopsy and prostatectomy GSs was calculated through the kappa statistic [15]. The kappa statistic corrects for agreement expected by chance: kappa values < 0 indicates poor agreement; 0 to < 0.20 indicate slight agreement; 0.21 to < 0.40 indicate fair agreement; 0.41 to < 0.60 indicate moderate agreement; 0.61 to < 0.80 indicate substantial agreement; and 0.81 to ≤ 1.0 indicate almost perfect agreement [16]. We calculated kappa for the individual GS (2 to 10) and for categories of GS (≤6, 7, 8–10). Rates of undergrading of GS categories were calculated and with logistic regression we assessed determinants of GS categories undergrading. We used a case–control approach considering as cases those patients whose biopsy GS was lower than the prostatectomy GS, and as controls everybody else. In the multivariate analysis we used the back step procedure, entering all the variables statistically significant in univariate analysis at the same time.

All data analysis was conducted at the Geneva Cancer registry. The Registry has general registry approval by the Swiss Federal Commission of Experts for professional secrecy in medical research (Commission d’experts pour le secret professionnel en matière de recherche medical). This approval permits cancer data collection and its use for research purposes.

## Results

The prostate cancer patients were on average 63 (±6.3) years old at diagnosis. For 242 (65%) patients, the PSA value at diagnosis was below 10 ng/ml; the clinical stage was classified as T1-T2a for 73% of patients. The median prostate volume was 36.6 cc (range 4–166) and the median number of biopsy cores was 10 (range 2–22). Thirty percent of patients had a biopsy by a physician performing less than 10 biopsies in the three-year study period. The median delay between biopsy and prostatectomy was 84 days (range 7–412).

**Figure 1:**
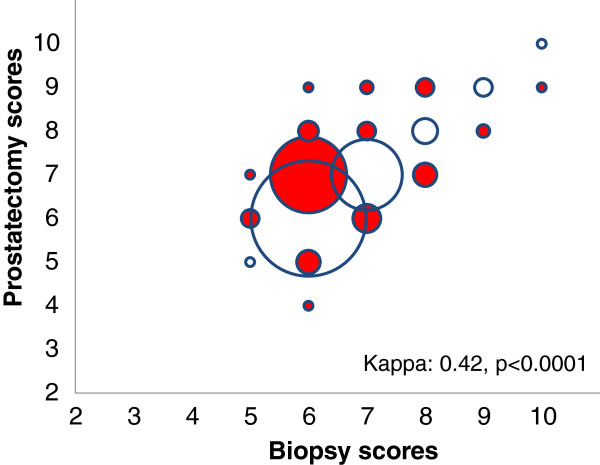
**Concordance between biopsy and prostatectomy individual Gleason scores.** Empty circles represent concordant scores between biopsy and prostatectomy; red filled circles represent discordant scores. The size is proportional to the number of cases falling in each combination.

Figure 1 shows the concordance between biopsy and prostatectomy individual GSs. An exact match was observed for 67% of patients (n = 248) while for 26% (n = 95) the GS was underestimated by the biopsy; in 7% of patients, it was overestimated. The kappa statistic was 0.42, indicating a moderate agreement. When considering the categories of GS (≤6, 7, 8–10) the patients understaged by the biopsy were 87 (23%) while the GS matched for 267 patients (72%). The kappa for the categories of GS was 0.48 (p < 0.0001), also indicating a moderate agreement. Over 30% of patients classified as Gleason score six or less from the biopsy, were Gleason score seven or more according to the prostatectomy.

The concordance significantly differed across categories of needle cores, number of biopsies performed by the operator and time between the biopsy and the prostatectomy. The kappa statistic for the patients who had nine or less biopsy cores was 0.35 (p < 0.001) and the proportion of concordance was 62%. For the patients who had 10 or more biopsy cores the kappa statistic was 0.49 (p < 0.001) and the proportion of concordance increased to 72%. The kappa statistic between biopsy and prostatectomy GS was 0.34 (p < 0.001) for the patients who had their biopsy performed by an operator with less than 30 procedures during the study period, and 0.44 (p < 0.001) if the operator performed 30 or more procedures. The kappa statistic for patients who had their prostatectomy within the median value (84 days, 2.7 months) from the biopsy was 0.54 (p < 0.001), the statistic for the group who had a longer delay was 0.31 (p < 0.001).

In the univariate logistic regression analysis, the variables associated with biopsy undergrading (cases = 95) were age, sector of care of the first treatment, clinical stage, number of biopsy cores, prostate volume, number of procedures performed by the biopsy operator, and the delay between the biopsy and the prostatectomy (Table 1). In the multivariate analysis the variables independently associated with undergrading selected for the model were age, with a 4% increase by each extra year of age (OR: 1.04, 95% CI: 1.00-1.09; p = 0.038), clinical stage (OR: T3-T4 vs. T1-T2: 1.81, 95% CI: 1.06-3.10; p = 0.030), number of cores (OR ≤9 cores vs. 10+: 1.93, 95% CI: 1.16-3.22; p = 0.011), and delay between biopsy and prostatectomy, with 1% increase by each extra day of delay between the 2 procedures (OR: 1.01, 95% CI: 1.00-1.01, p = 0.018) (Table 1).

**Table 1:** Determinants of Gleason score undergrading between biopsy and prostatectomy

	**Total**	**Cases**	**Controls**	**Univariate OR**^**1 **^**(95% CI)**	**p-value**	**Multi-adjusted OR**^**1,2 **^**(95% CI)**	**p-value**
	**(n = 371)**	**(n = 95)**	**(n = 276)**				
**Age at diagnosis** years (in continuous )				1.04 (1.00-1.08)	0.049	1.04 (1.00-1.09)	0.038
**Socioeconomic status**							
High	128	31	97	1.00			
Middle	155	43	112	1.20 (0.70-2.05)	0.502		
Low	82	20	62	1.01 (0.53-1.93)	0.977		
Unknown	6	1	5	0.63 (0.07-5.56)	0.674		
**Sector of care**							
Private	235	48	187	1.00			
Public	136	47	89	2.06 (1.28-3.31)	0.003		
**PSA value**							
<10	242	63	179	1.00			
10-20	39	14	25	1.59 (0.78-3.25)	0.203		
>20	12	4	8	1.42 (0.41-4.88)	0.577		
Unknown	78	14	64	0.62 (0.33-1.19)	0.149		
**Clinical stage**							
T1-T2	272	61	211	1.00		1.00	
T3-T4	93	33	60	1.90 (1.14-3.17)	0.014	1.81 (1.06-3.10)	0.030
Tx	6	1	5	0.69 (0.08-6.03)	0.739	0.58 (0.06-5.37)	0.628
**Number of biopsy cores**							
≤ 9	175	56	119	2.02 (1.24-3.28)	0.005	1.93 (1.16-3.22)	0.011
10 +	185	35	150	1.00		1.00	
Unknown	11	4	7	2.45 (0.68-8.83)	0.171	2.65 (0.70-10.0)	0.151
**Prostate volume** cm3 (in continous)				0.99 (0.97-1.00)	0.031		
**Number of procedures performed by the biopsy operator**							
1-29	110	37	73	1.75 (1.07-2.87)	0.026		
30+	254	57	197	1.00			
Unknown	7	1	6	0.58 (0.07-4.88)	0.613		
**Delay between biopsy and prostatectomy** days (in continous)				1.01 (1.00-1.01)	0.004	1.01 (1.00-1.01)	0.018

1) OR: Odds Ratio derived from logistic regression considering as cases patients whose biopsy Gleason score was lower than the prostatectomy Gleason score and as controls everybody else, 2) Simultaneously adjusted for age, pathological stage, number of biopsy cores and delay between the two procedures.

## Discussion

This study shows that in Geneva, the concordance between the GSs of biopsy and those of prostatectomy is moderate, and lies within the current standards. Our study also confirms that a lower number of biopsy cores is an important and independent determinant of biopsy undergrading.

The finding of a 67% exact match in grading between biopsy and prostatectomy observed in Geneva is similar to the proportion found in other studies. Muntener et al. and Rajinikanth et al. both found exact match between biopsy and prostatectomy GS categories as in 69% of patients [4,8]. In particular the latter found that the exact match improved from 58% in 1992–1996 to 75% in 2002–2006, which represents a period closer to our study period [8].

As reported by other authors, we found that a higher number of biopsy cores is associated with a decreased risk of undergrading [10,17]. In our study, the proportion of exact matching improved significantly, from 62% to 72%, when the patients had ten or more cores compared with those who had nine or less, reinforcing the 2008 EAU guidelines recommendations of a minimum of ten systematic cores per biopsy [12]. However, the most recent EAU guidelines reduced to eight the minimum number of biopsy cores recommended, suggesting not to exceed twelve, with adaptations according to the prostate volume [13], with the likely intent to keep a high validity and at the same time reduce the patient’s discomfort and the number of biopsy complications. Serious complications may, in fact, occur after a prostate biopsy, infection being one of the most serious ones [18].

As opposed to the report from Antunes et al. [10], we found that the effect of the number of biopsy cores on the risk of undergrading did not depend on the radical prostatectomy specimen volume in multivariate analysis.

In our study, older age and a higher clinical stage of the disease at diagnosis were also predictors of biopsy undergrading, consistently with previous investigations [9,11,17,19,20]. This could be linked to increasing intra-tumoral heterogeneity of prostate cancer among men with advanced age and advanced clinical stage, which could affect clinical estimates of the grade of malignancy [21-23].

We found that the concordance was much higher among the group of patients with a shorter delay between the biopsy and the prostatectomy (<= 84 days, kappa = 0.54) than in the group with a delay longer than 84 days (kappa = 0.31). For this reason we adjusted our multivariate model by the delay between the two procedures. Consistently with our results, Kvale et al. found that upgrading increased with increasing interval from biopsy to radical prostatectomy [17]. Delay between biopsy and surgery is more likely to have occurred in lower grade tumors and may well explain the higher discordance with the final specimen. However, a histological dedifferentiation of the tumor over time has been demonstrated by some authors, but its association with disease progression is not clear [21-23].

This study has some limitations. Most importantly, both biopsy and prostatectomy specimens were examined by different laboratories, leading to possible inter-observer variability common in GS interpretation, particularly for less experienced pathologists [24]. In our study, however, two main laboratories analyzed approximately 92% of all prostate biopsies and approximately 70% of all prostate surgical samples of prostate cancer cases, which suggests a substantial experience by the examining laboratories, although we cannot rule out variability between observers. Furthermore, we found no significant difference of concordance between biopsy and prostatectomy GS by the average number of biopsy analyzed by each pathologist (data not shown).

The strength of this study is its population-based approach and that it covers a recent period of time, i.e. 2004–2005. The period chosen overlaps with a refinement of the Gleason grading system promoted in 2005 by the International Society of Urological Pathology consensus conference (ISUP) [25]. However, we estimate that this revision had a negligible impact on our results. In fact, contrary to other studies which show after the ISUP revision a tendency towards a higher Gleason prognostic due mainly to an upgrading of the secondary pattern, we did not observe any difference in the proportion of high *vs.* low grade cancers by year of diagnosis [26-28]. Similarly, we did not detect a difference in neither the primary nor the secondary Gleason pattern, for both the biopsy and the prostatectomy (p-value Pearson chi–square pattern1 and pattern 2 biopsy: 0.856 and 0.663; pattern1 and pattern2 prostatectomy: 0.983 and 0.679, respectively). Biopsy GS is an important outcome predictor of prostate cancer, and especially so for patients treated conservatively. Of particular clinical relevance is the biopsy undergrading of patients from grade seven or more to grade six or less, as these patients would move from a high/intermediate to a low risk group with a high probability of receiving a less aggressive treatment approach. In our study, there were 147 patients with T1-T2 prostate cancer stage, PSA < 10 ng/ml, and a biopsy Gleason grade <7 who would have been ideal candidates for active surveillance. After the prostatectomy the Gleason was upgraded to a grade > =7 for 36 of these patients. Thus, in this population-based sample approximately 10% of patients with high-risk disease would have been undertreated. These results support the active surveillance protocols to rebiopsy patients one year after their diagnosis to reduce the proportions of understaged patients to approximately 5% [29].

## Conclusions

In conclusion, this study confirms that number of biopsy cores impacts the concordance between Gleason of biopsy and prostatectomy. Several actions can be taken to optimize concordance of prostate cancer grading between needle biopsy and the prostatectomy specimen. Most importantly, adequate training of urologists in performing biopsies has the potential not only to optimize diagnostic accuracy but to minimize the patient’s discomfort and morbidity as well. Extended needle biopsy protocols, with at least ten cores, improve cancer detection rates and the concordance of biopsy results with the prostatectomy findings. All pathologists evaluating prostate tissues should be systematically trained in Gleason grading. Furthermore, as recently recommended by the National Institute for Health and Clinical Excellence guidelines [1], results of all prostate biopsies should be reviewed by a urological cancer multidisciplinary team. Men who have selected active surveillance as treatment option should have a repeat biopsy after a review by this team of the risk characteristics, including life expectancy, PSA level, digital rectal examination, and prostate volume. Use of modern imaging devices, such as three dimensional ultrasound during needle biopsy, could further help to improve diagnostic accuracy prior to clinical decision making.

## Abbreviations

EAU: European Association of Urology; GSs: Gleason scores; GS: Gleason score; PSA: Prostate-specific antigen.

## Competing interests

All the authors declare that they have no financial competing interests.

## Authors’ contributions

ER participated in the conception and design, data analysis, interpretation, drafting and supervision of the manuscript. RS performed the data acquisition and statistical analysis. CI participated in the data interpretation and revision of the manuscript. RM participated in the data interpretation and revision of the manuscript. MFP performed the data acquisition. DW made a revision of the manuscript. RZ made a revision of the manuscript. INC performed the data acquisition. CB participated in the conception and design, data analysis, interpretation, drafting and final approval of the version. All authors read and approved the final manuscript.

## Pre-publication history

The pre-publication history for this paper can be accessed here:

http://www.biomedcentral.com/1471-2490/13/19/prepub
